# Biofabrication of Cell-Derived Nanovesicles: A Potential Alternative to Extracellular Vesicles for Regenerative Medicine

**DOI:** 10.3390/cells8121509

**Published:** 2019-11-25

**Authors:** Nazma F. Ilahibaks, Zhiyong Lei, Emma A. Mol, Anil K. Deshantri, Linglei Jiang, Raymond M. Schiffelers, Pieter Vader, Joost P.G. Sluijter

**Affiliations:** 1 Laboratory of Experimental Cardiology, Department of Cardiology, University Medical Center Utrecht, 3584 CX Utrecht, The Netherlands; n.f.ilahibaks-2@umcutrecht.nl (N.F.I.); z.lei@umcutrecht.nl (Z.L.); E.A.Mol-5@umcutrecht.nl (E.A.M.); pvader@umcutrecht.nl (P.V.); 2 Department of Clinical Chemistry and Hematology, University Medical Center Utrecht, 3584 CX Utrecht, The Netherlands; anildeogarh@gmail.com (A.K.D.); L.Jiang-2@umcutrecht.nl (L.J.); R.Schiffelers@umcutrecht.nl (R.M.S.); 3 Circulatory Health Laboratory, Regenerative Medicine Center, University Medical Center Utrecht, University Utrecht, 3584 CX Utrecht, The Netherlands

**Keywords:** extracellular vesicles, biofabrication, cell-derived nanovesicles, therapeutic delivery, regenerative medicine

## Abstract

Extracellular vesicles (EVs) are mediators of intercellular communication by transferring functional biomolecules from their originating cells to recipient cells. This intrinsic ability has gained EVs increased scientific interest in their use as a direct therapeutic in the field of regenerative medicine or as vehicles for drug delivery. EVs derived from stem cells or progenitor cells can act as paracrine mediators to promote repair and regeneration of damaged tissues. Despite substantial research efforts into EVs for various applications, their use remains limited by the lack of highly efficient and scalable production methods. Here, we present the biofabrication of cell-derived nanovesicles (NVs) as a scalable, efficient, and cost-effective production alternative to EVs. We demonstrate that NVs have a comparable size and morphology as EVs, but lack standard EV (surface) markers. Additionally, in vitro uptake experiments show that human fetal cardiac fibroblast, endothelial cells, and cardiomyocyte progenitor cells internalize NVs. We observed that cardiac progenitor cell-derived NVs and EVs are capable of activating mitogen-activated protein kinase 1/2 (MAPK1/2)-extracellular signal-regulated kinase, and that both NVs and EVs derived from A431 and HEK293 cells can functionally deliver Cre-recombinase mRNA or protein to other cells. These observations indicate that NVs may have similar functional properties as EVs. Therefore, NVs have the potential to be applied for therapeutic delivery and regenerative medicine purposes.

## 1. Introduction

Extracellular vesicles (EVs) are cell-derived nanoparticles consisting of a lipid bilayer encapsulating cytosolic content from their cell of origin. EVs can exchange information between cells by delivering biomolecules, e.g., proteins, nucleic acids, or lipids, from their parent cells to target cells [1]. EVs′ endogenous origin, low immunogenicity, intrinsic targeting abilities, and biocompatibility are potential advantages features compared to conventional drug delivery systems such as liposomes, polymers, and gold particles [2,3,4].

EVs are also considered as a cell-free therapy for regenerative medicine since various studies have shown that progenitor cells exert their therapeutic reparative benefit via a paracrine mechanism in which EVs play a fundamental role [2,3,4,5,6,7,8,9,10,11]. In cardiovascular disease, human fetal cardiac progenitor cell (CPC)-derived EVs stimulate pro-angiogenic effects and improve cardiac function upon injury [12,13]. Therefore, CPC-EVs are an attractive therapeutic candidate to stimulate cardiac repair and regeneration, since the heart has a very poor endogenous regenerative capacity [14,15,16,17,18]. 

Despite EVs′ potential, their application is restrained due to the lack of highly efficient, scalable, and cost-effective production and isolation methods [19]. Large amounts of cells and culture medium, as well as time-consuming and labor-intensive isolation and purification processes, are required to obtain substantial quantities of EVs [20,21]. Various approaches have been investigated to augment the production of EVs [21,22,23,24,25]. However, these studies either do not show data indicating whether their approach yields considerable amounts of EVs, save time in their production or isolation processes, or demonstrate functional delivery of EVs. 

**Figure 1:**
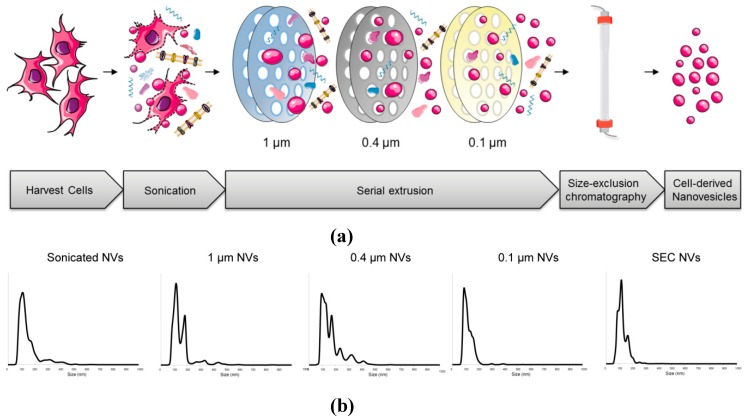
Establishment of the biofabrication of nanovesicles (NVs) in cardiac progenitor cells (CPCs). (**a**) Schematic illustration of NV generation and purification. (**b**) NTA showed that the extrusion process through filters yielded a homogenous NVs population with a ~50–200 nm peak diameter. The size of the NVs became more homogenous after each step. These figures are representative results of at least four independent experiments.

Hence, this study aimed to investigate the spontaneous self-assembly of cell-derived vesicles (NVs), so-called biofabrication of NVs, as an alternative production method to make EV mimetics. During NV biofabrication, cells are subjected to sonication followed by sequential extrusion through microfilters to yield nanosized vesicles (Figure 1a). We hypothesized that during the NV biofabrication process, the membrane compositions of parental cells would remain, while their cytosolic content would partially be encapsulated in the lumen of NVs. 

We found that biofabrication of NVs is a scalable and efficient alternative of EV production to obtain a high yield of cell-derived nanosized vesicles. We also demonstrated that NVs are capable of transferring functional biological material to recipient cells and can activate intracellular pro-survival signaling pathways. To the best of our knowledge, this is the first report on biofabrication of CPC-NVs that demonstrates the induction of phosphorylation of mitogen-activated protein kinase 1/2 (MAPK1/2)-extracellular signal-regulated kinase (ERK1/2) and the active delivery of Cre-recombinase mRNA/protein into target cells. These observations indicate that NVs have the potential to be further developed as a potential alternative for CPC-EVs in regenerative medicine or be engineered for drug delivery purposes. 

## 2. Materials and Methods

### 2.1. Cell Culture

Human embryonic kidney cells 293T (HEK293T), 293FT cells (HEK293FT), and human fetal cardiac fibroblasts (CF) were cultured in Dulbecco′s Modified Eagle Medium (DMEM) (41965039, Gibco, Paisley, Scotland, UK) supplemented with 10% (*v*/*v*) fetal bovine serum (FBS) and 1% (*v*/*v*) penicillin-streptomycin (P/S). Human fetal heart tissue was obtained by individual permission via a standard written informed consent procedure following the Declaration of Helsinki and approval of the ethics committee of the Leiden University Medical Center (LUMC), The Netherlands. The subsequently obtained human cardiac-derived progenitor cells were provided anonymously via an MTA from LUMC, The Netherlands. CPC and human microvascular endothelial cells (HMEC-1) were obtained and cultured in appropriate cell culture media, as previously described [13,26,27]. Human umbilical vein endothelial cells with a cytosolic green fluorescent protein (HUVEC-GFP) were obtained and cultured, as described previously [13]. A431-Cre, HEK293FT-Cre donor cells, and T47D-stoplight reporter cells were cultured in DMEM (41965039, Gibco, Paisley, Scotland, UK) supplemented with 10% FBS (*v*/*v*), 1% P/S (*v*/*v*), and 2 μg/mL puromycin. All cells were maintained in a 5% CO_2_ atmosphere at 37 °C. 

#### HEK293FT-Cre Donor Cell Line

HEK293FT-Cre stable cell lines were made via lentiviral transduction. Lentiviral envelopes were created with VSV-G and CMV helper plasmid lipofectamine-mediated transfection (L3000008, Lipofectamine^®^ 3000 Transfection Reagent, Invitrogen Corp., Carlsbad, CA, USA)) in HEK293FT cells. In a 0.1% gelatin-coated 6-well plate, 500,000 HEK293FT cells/well were plated out and incubated O/N. After 24 h, the medium was changed to DMEM (41965039, Gibco, Paisley, Scotland, UK) and supplemented with 10% (*v*/*v*) FBS. For lipofectamine transfection, a DNA sample mixture containing VSV-G (0.5 µg), CMV (1 µg) and Cre plasmid (1 µg) was mixed in NaCl (100 µL, 150 mM, pH 7.1) together with 5 µg P3000™ Reagent. Subsequently, Lipofectamine™ 3000 Reagent (5 µg) was added to the DNA mixture and incubated together for 15 min at room temperature (RT). The DNA-lipid complex was added to the cells and incubatedn a 5% CO_2_ environment at 37 °C. After 5–6 h, the DNA-lipid complex containing media was aspirated and replaced with (DMEM) (Gibco, 41965039, Paisley, Scotland, UK) supplemented with 10% (*v*/*v*) FBS and 1% (*v*/*v*) P/S. After 48 h, the supernatant was collected and centrifuged at 2000× *g* for 5 min. The viral containing supernatant was added to HEK293FT cells (100,000 cells/well in a 24-well plate seeded the day before), with polybrene. After 48 h, the viral medium was replaced with selection medium, i.e., DMEM supplemented with 10% (*v*/*v*) FBS, 1% (*v*/*v*) P/S, and 2 μg/mL puromycin.

### 2.2. NV Biofabrication

Firstly, 1–5 × 10^7^ cells were harvested with 0.25% trypsin–EDTA (SLB57926) and washed with Phosphate Buffered Saline (PBS) (10010023, Gibco, Paisley, Scotland, UK). To disrupt the cells, they were resuspended in 5 mL PBS (10010023, Gibco, Paisley, Scotland, UK) and sonicated. Subsequently, the sonicated sample was recovered, transferred to the LiposoFast LF-50 (Avestin, York, UK) and extruded under compressed nitrogen through Nuclepore Track-Etch Membrane Whatman polycarbonate filters in a serial manner, i.e., starting with a 1.0 µm (110610, Whatman, Maidstone, UK) followed by 0.4 µm (110607, Whatman, Maidstone, UK), and finally four times through a 0.1 µm Whatman^®^ Anodisc inorganic filter membrane (6809-6012,, Whatman, Maidstone, UK),) to yield a homogenous population size. The crude NV sample was loaded on the S400 high prep column (GE Healthcare, Uppsala, Sweden) using AKTAStart (GE Healthcare) with a 280 nm UV flow cell at 4 °C. The NVs-containing fractions were combined, filtered (0.45 µm), and concentrated with an Amicon Ultra-15 Centrifugal Filter with an Ultracel-100 membrane (UFC910024, Merck, Darmstadt, Germany). See Figure 1a for a schematic illustration of the process. 

### 2.3. EV Purification

EVs were isolated by culturing 70–90% confluent HEK293FT-Cre and A431-Cre in Opti-MEM I Reduced Serum Media (31985070, Gibco, Paisley, Scotland, UK) supplemented with 1% P/S. Also, CPC cells were cultured in M199 medium (3115022, Gibco, Paisley, Scotland, UK) as described before [28]. After 24 h, the conditioned medium was collected and centrifuged at 2000× *g* for 15 min to remove cell debris. The supernatant was collected, filtered (0.45 µm), and concentrated with an Amicon Ultra-15 Centrifugal Filter with an Ultracel-100 membrane (UFC910024, Merck, Darmstadt, Germany). Subsequently, the concentrated conditional medium was loaded on an S400 high prep column similarly as for crude NVs. After size-exclusion chromatography (SEC), the EV-containing samples were collected, filtered (0.45 µm), and concentrated with an Amicon Ultra-15 Centrifugal Filter with an Ultracel-100 membrane (UFC910024, Merck, Darmstadt, Germany). 

For the Cre-loxP functionality experiment, NVs and EVs were isolated from A431-Cre and HEK293FT-Cre donor cells. These vesicles were purified accordingly to generally accepted ultracentrifugation protocol due to efficiency reasons, i.e., all samples were purified at the same time. Conditioned medium containing EVs and the crude NVs samples were centrifuged at 2000× *g* for 15 min at 4 °C to remove dead and floating cells. Subsequently, the supernatant was centrifuged at 10,000× *g* for 30 min at 4 °C to remove small vesicles and small cell debris. Finally, the supernatant was centrifuged at 100,000× *g* for 60 min at 4 °C to pellet the vesicles.

### 2.4. Nanoparticle Tracking Analysis

The size and particle concentration of EVs and NVs were assessed with nanoparticle tracking analysis (NTA) (Nanosight NS500, Malvern Panalytical Ltd., Malvern, UK). EVs and NVs were dispersed in PBS and measured in triplicate with individual measurements of 30 s at camera level 14. The analysis was performed with NTA software 3.3 with a minimal track length of 10, detection threshold of 5, and screen gain of 1. 

### 2.5. Western Blot

For Western Blot analysis of vesicle protein surface markers, CPC cell lysate (CL) were dispersed in cOmplete™ Lysis-M EDTA-free (4719964001, Roche Applied Science, Mannheim, Germany) according to the manufacturer’s guidelines, CPC-EVs and CPC-NVs were dispersed in RIPA buffer. Protein levels were measured with microBCA (23235, ThermoFisher Scientific, Rockford, IL, USA) and normalized to 1 µg per sample. Protein samples used for CD63 detection were boiled at 70 °C for 10 min. For the detection of other proteins, samples were denatured with NuPAGE™ Sample Reducing Agent (10×) (NP0004, Invitrogen Corp., Carlsbad, CA, USA) and boiled at 70 °C for 10 min. Samples were loaded on Bolt™ 4–12% Bis-Tris Plus Gel (NW04125BOX, ThermoFisher Scientific, Rockford, IL, USA) at 165 V for 60 min and transferred to PVDF membranes (IPVH00010, Merck, Darmstadt, Germany). Membranes were blocked with 5% Bovine Serum Albumin (BSA) in Tri-Buffered Saline (TBS) for 1 h at RT. Subsequently, membranes were incubated with primary antibodies against Alix (177840, Abcam, Cambridge, UK), CD81 (B-11; sc-166029, Santa Cruz Biotechnology, Inc., Santa Cruz, CA, USA), Flotillin-1 antibody (ab41927, Abcam, Cambridge, UK), Calnexin (GTX101676, GeneTex, Irvine, CA, USA), β-actin (A5441, Sigma-Aldrich, Saint Louis, MO, USA), or CD63 (8219, Abcam, Cambridge, UK). Subsequently, membranes were washed and incubated for 1h with appropriate secondary antibodies Goat Anti-Mouse Immunoglobulins/HRP (P0447, Dako, Santa Clara, CA, USA) or Goat Anti-Rabbit Immunoglobulins/HRP (P0448, Dako, Santa Clara, CA, USA). Proteins were visualized with chemiluminescent peroxidase substrate (CPS1120, Sigma-Aldrich, Saint Louis, MO, USA).

### 2.6. Transmission Electron Microscopy

To compare the morphology of EVs and NVs, transmission electron microscopy was performed. Carbon film copper grids (75–200 mesh) were freshly coated with the carbon coater (Edward) and 10 µL of CPC-EVs and CPC-NVs samples were placed on parafilm. The carbon-coated grids were placed on top of the samples and incubated for 15 min at RT. Unbound vesicles were removed with PBS followed by fixation of the bound vesicles with 1% glutaraldehyde in PBS for 15 min at RT. PBS was removed by washing the grids on 0.2 µm filtered demi water. The vesicles were stained with 2% uranyl oxalate in 0.15 M oxalate (pH 7.0) for 10 min at RT, followed by removal of excess stain solution with filter paper (1001090, Whatman, Maidstone UK) and MilliQ. Subsequently, the grids were incubated with 1.8% methylcellulose and 0.4% uranyl acetate for 10 min on ice. Excess fluid was removed and the grids were dried for 1 h at RT. CPC-EVs and CPC-NVs were visualized with a JEOL 3 microscope. 

### 2.7. Uptake Experiment 

One-hundred thousand cells/well of hfCF, CPC, HMEC, and HUVEC-GFP were plated in a 24-well plate containing 0.1% gelatin-coated cover glasses and incubated O/N. HEK293T-NVs were generated, purified with SEC, and stained with 10 µM PKH-26 (PKH26GL, Sigma-Aldrich, Saint Louis, MO, USA) for 10 min at RT. Excess PKH-26 was removed by purification of the sample on an XK16 column using AKTAStart at 4 °C. The PKH-26 labeled HEK293T NVs containing fractions were pooled, filtered (0.45 µm), and then concentrated with a 100 kDa MWCO Amicon spin filter (Merck). Subsequently, the amount of NVs was measured with a NTA. Then, 2 × 10^10^ NVs were added individually to hfCF, CPC, HMECs, and HUVECS-GFP and incubated for 24 h in a 5% CO_2_ environment at 37 °C. After 24 h, the medium was aspirated, the cells were washed with PBS and fixated with 4% paraformaldehyde (PFA) for 10 min and washed three times with PBS for 5 min at RT. The nuclei were stained with Hoechst, and the cover glasses were transferred to microscope slides on Fluoromount-G (0100-01, Southern Biotech, Birmingham, AL, USA). HEK293T-NVs uptake was visualized with Olympus BX53 microscope (magnification 20×) and confocal Zeiss LSM700 microscope (magnification 63×).

### 2.8. Functional Delivery of Cre by NVs and EVs

One-hundred thousand T47D-stoplight reporter cells/well were plated in a 24-well plate with 0.1% gelatin-coated cover glasses and incubated O/N. Then, 0.25 µg TAT-CRE Recombinase (SCR508, Merck) or 0.25 µg A431-Cre- and HEK293FT-Cre-derived EVs and NVs were added to T47D-stoplight reporter cells. After 72 h, the medium was aspirated, the cells were washed with PBS, fixated with 4% PFA for 10 min at RT, and washed three times with PBS for 5 m. Subsequently, the fixated cells were stained with Hoechst. The functionality of A431-Cre- and HEK293FT-Cre-derived NVs was assessed by the presence of eGFP^+^ T47D-stoplight reporter cells after treatment, compared to TAT-CRE Recombinase and EVs derived for the same cell-lines, using a Zeiss LSM700 microscope (magnification 63×, with 405, 488, and 555 nm lasers used). 

### 2.9. Activation of ERK Signaling by CPC-EVs and -NVs

One-hundred thousand HMEC cells/well were plated in a 0.1% gelatin-coated 48-well plate and incubated O/N. HMECs were starved in MCDB-131 medium (Gibco) for 3 h and stimulated with 3 × 10^10^ CPC-EVs or CPC-NVs for 30 m. To assess pERK1/2 and ERK1/2 protein levels after CPC-NVs and CPC-EVs stimulation, HMEC cell lysates were obtained by suspending cells in cOmplete™ Lysis-M EDTA-free (4719964001, Roche Applied Science, Mannheim, Germany). Subsequently, protein levels were determined by microBCA assay (23235, ThermoFisher Scientific, Rockford, IL, USA) and normalized before loading the samples on Bolt™ 4–12% Bis-Tris Plus Gel (NW04125BOX, ThermoFisher Scientific, Rockford, IL, USA). Samples were run at 165V for 50 min and transferred to PVDF membranes (IPVH00010, Merck, Darmstady, Germany). Membranes were blocked for 1 h with 5% BSA in TBS and incubated with primary antibodies phospho-p44/42 MAPK (pERK1/2) (, 9101S, Cell Signaling, Beverly, CA, USA) and p44/42 MAPK (Erk1/2) (9102S, Cell Signaling, Beverly, CA, USA). Subsequently, membranes were washed with TBS and incubated with Goat Anti-Rabbit Immunoglobulins/HRP (P0448, Dako, Santa Clara, CA, USA) secondary antibody. Proteins were visualized with chemiluminescent peroxidase substrate (CPS1120, Sigma-Aldrich, Saint Louis, MO, USA).

### 2.10. Comparative Analysis NV and EV Production 

Vesicle number and protein concentration of NVs and EVs recovered from 1 × 10^7^ CPC were determined with NTA and microBCA (23235, ThermoFisher Scientific, Rockford, IL, USA), respectively. The total number of particles and protein content was calculated by multiplying the final volumes of NVs or EVs by the measured particle and protein concentrations. 

### 2.11. Statistical Analysis

All statistical analysis was performed with GraphPad Prism 7 using one-way ANOVA with Bonferroni correction for multiple comparisons. Data is presented as mean ± SEM. A *p*-value < 0.05 was considered as significant difference. 

## 3. Results

### 3.1. Production 

#### 3.1.1. Process Characterization

To produce NVs, cells were sonicated, sequentially extruded through membranes with decreasing pore size (subsequently 1, 0.4, and 0.1 µm) using compressed nitrogen (10–40 bar), which was followed by size-exclusion chromatography (SEC) purification (Figure 1a) [28]. NTA showed that the nonhomogenous populations yielded by sonication are formed into a homogenous NV peak-diameters between ~50–200 nm through sequential extrusion through microfilters (Figure 1b). 

#### 3.1.2. Characterization of CPC-NVs and CPC-EVs 

**Figure 2:**
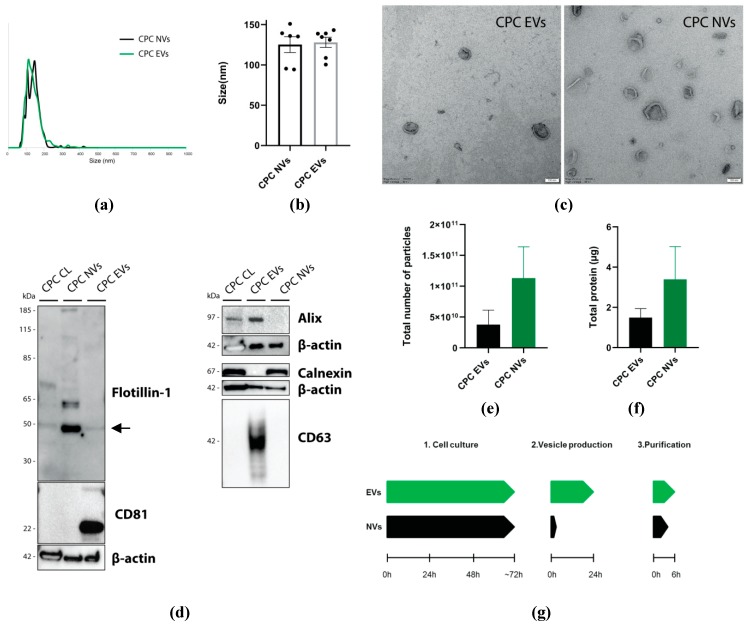
Comparative analysis of CPC-EVs and CPC-NVs characteristics and production process. NTA analysis showed that CPC-EVs and CPC-NVs have a comparable peak diameter between (**a**) ~50–200 nm, and (**b**) a mean diameter ± 126 nm. (**c**) Transmission electron microscopy showed a similar ‘cup-shaped’ morphology for both CPC-EVs and NVs. Scale bar = 100 nm (**d**) Western blot analysis of 1 µg CPC cell lysate (CL), CPC-NVs and CPC-EVs show that CPC-NVs are enriched with flotillin-1 and calnexin. In contrast, CPC-EVs are enriched with EV protein markers CD81, CD63, and Alix. (**e**) Total number of particles and (**f**) total protein content of CPC-EVs and CPC-NVs derived from 1 × 10^7^ CPC show that NVs are produced in a higher quantity than EVs when derived from the same cell density (*n* = 2). (**g**) Schematic overview of the production and purification timeline of EVs and NVs shows that NV production is more efficient compared to EV production. Data expressed as mean ± SEM. EVs = extracellular vesicles.

CPC-NVs and CPC-EVs were characterized for size, morphology, and protein surface markers. NTA analysis of purified CPC-EVs and CPC-NVs showed a comparable size distribution between ~50–200 nm, with a mean diameter ± 126 nm (Figure 2a,b). Transmission electron microscopy showed a ‘cup-shaped′ morphology of CPC-NVs, which was comparable to the morphology of CPC-EVs (Figure 2c). Western blot analysis demonstrated that CPC-NVs are enriched for integral caveolae-associated membrane protein flotillin-1, an integral protein of the endoplasmic reticulum calnexin and β-actin. In contrast, CPC-EVs are enriched for tetraspanins CD81 and CD63 and the multivesicular body protein Alix (Figure 2d). Additionally, NTA of two individual CPC-NV preparations stored at −80 °C showed that CPC-NVs slightly increase in size and decrease in concentration over time (Figure S4).

To assess if NV biofabrication is more efficient than EV production, we determined the time needed to complete either production process and measured the quantity of NVs and EVs that were recovered from equal cell numbers. When vesicles were derived from 1 × 10^7^ CPC, the total yield in terms of the total number of particles and protein was higher for NV biofabrication compared to EV production (Figure 2e,f). To yield an approximately a similar amount of a total number of particles, CPC-EVs needs to be derived from 11-fold higher cell density compared to CPC-NVs (Figure S1). Also, the initial cell density prior to NV biofabrication determines the total number of particles recovered after SEC (Figure S2). Furthermore, NV biofabrication is more time-efficient and less laborious with respect to the production and purification phase (Figure 2g). Compared to EVs production, NV biofabrication requires less time, materials, and labor intensity for the upscaling of cell culture and eliminates a concentrating step to prepare conditioned medium for SEC. These observations show that after SEC, CPC-NVs are produced more efficiently (Figure 2e–g) and share a similar size-distribution and morphology as CPC-EVs (Figure 2b,c). However, CPC-NVs do not contain standard EV protein markers, indicating that they represent a different vesicle type. 

### 3.2. Functionality

#### 3.2.1. NVs Uptake 

**Figure 3:**
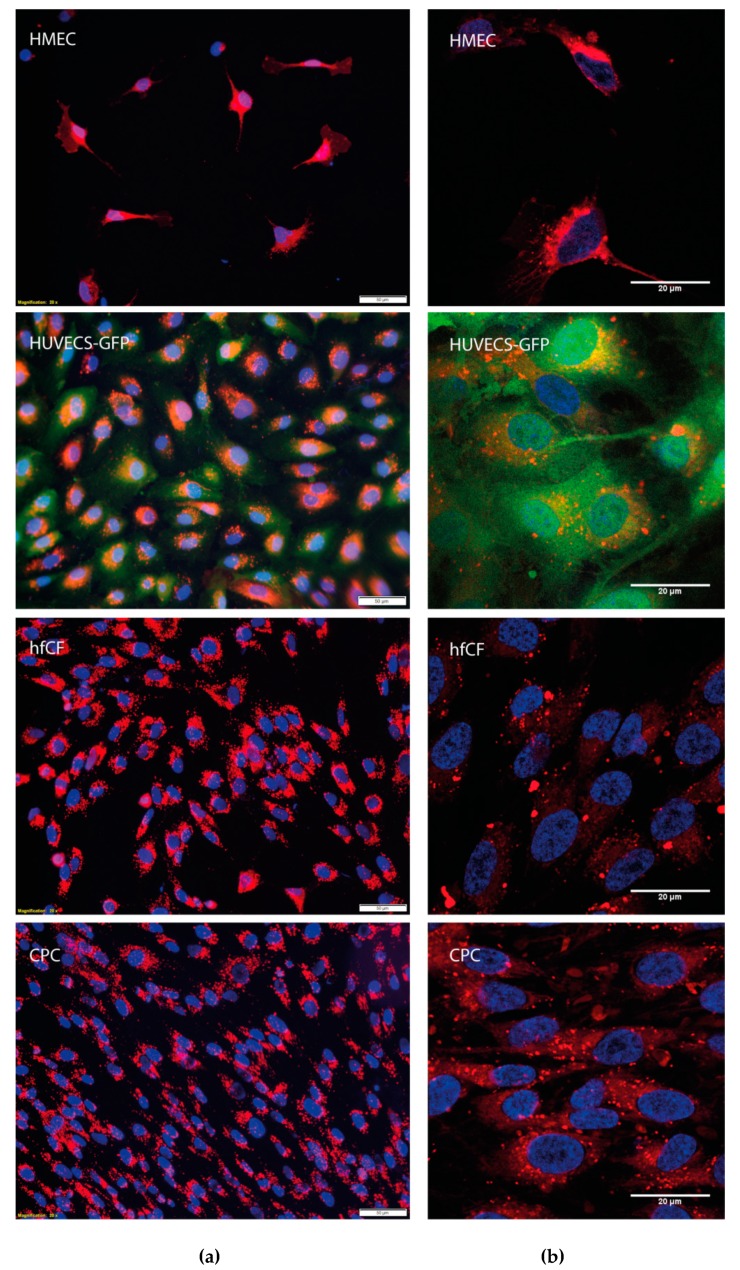
CPC-NV uptake by HMECs, HUVECS-GFP, hfCF, and CPCs. (**a**) Microscopic analysis by Olympus BX53 microscope. Scale bar = 50 µm. (**b**) Confocal microscopy by LSM Zeiss 700. Scale bar = 20 µm. (**a**) and (**b**) demonstrated internalization of PKH-26 positive NVs upon incubation of 2 × 10^10^ NVs with HMECs, HUVECS-GFP, hfCF, and CPC for 24 h. NVs = PKH-26 (red) and nucleus = Hoechst (blue).

To confirm that NVs could be internalized by cells, we labeled HEK293FT-NVs with a red fluorescent PKH-26 dye and incubated them for 24 h with different target cells, including two types of endothelial cells (HMECs and HUVECs-GFP), fibroblasts (hfCF), and cardiac progenitor cells (CPCs). The internalization of fluorescent-labeled NVs by endothelial and cardiac cells was confirmed by microscopy analysis (Figure 3a,b) and resulted in a peri-nuclear pattern, as we have seen before for EVs [13]. 

#### 3.2.2. NVs Functionally Deliver Biomolecules to Target Cells

**Figure 4:**
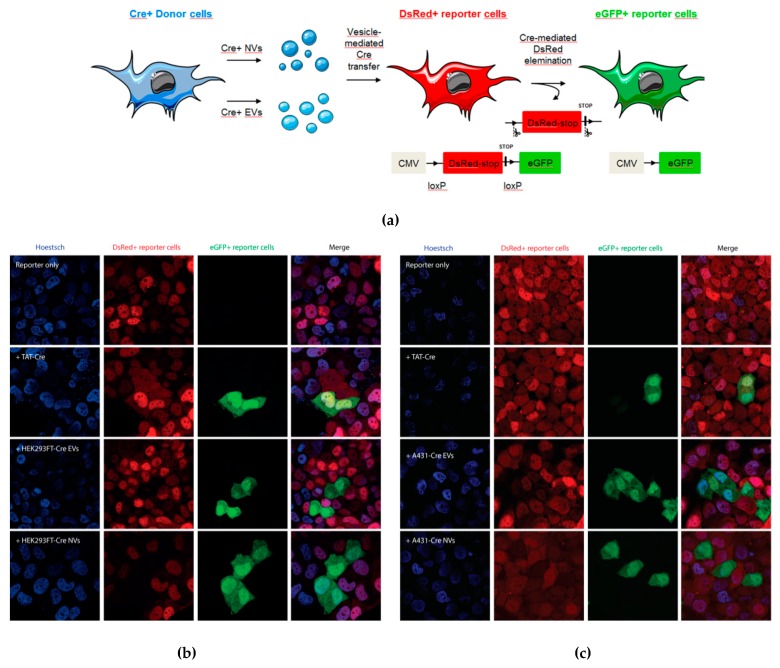
Cre+-EVs and Cre+-NVs transfer of Cre-recombinase mRNA or protein to T47D Cre-reporter cells. (**a**) Schematic illustration of Cre+ vesicle-transfer to reporter cells: DsRed+ reporter cells will be recombined to eGFP+ cells when the stop-codon is removed between indicated loxP sites. Visualization of functional effect induced by TAT-Cre recombinase, Cre+-EVs andCre+-NVs derived from HEK293FT-Cre (**b**), and A431-Cre donor cells (**c**).

To assess if NVs can deliver functional biomolecules to recipient cells, we used the Cre-loxP method to investigate the vesicle-mediated transfer of cargo to a T47D-stoplight reporter cell line. Upon delivery of Cre biomolecules (mRNA or protein) to the reporter cell line, DsRed+ reporter cells will be recombined to eGFP+ cells when the stop-codon is removed between loxP sites (Figure 4a). Confocal microscopy images confirmed the presence of eGFP+ reporter cells when TAT-Cre recombinase, Cre+-EVs, or Cre+-NVs were introduced (Figure 4b,c). Although the absolute number of eGFP+ reporter cells was low, we could reproducibly detect them for all these conditions. No eGFP+ reporter cells were observed when T47D-stoplight reporter cells were treated with vesicles derived from a non-Cre donor cell HEK293FT (data not shown). NTA characterization of Cre+-NVs and EVs showed that these vesicles share similar sizes (Figure S3a,b). These observations indicate that NVs and EVs have a similar size and are both capable of transferring functional biomolecules from donor cells to recipient cells. 

#### 3.2.3. CPC-NVs and EVs Induce Phosphorylation of ERK1/2 

**Figure 5:**
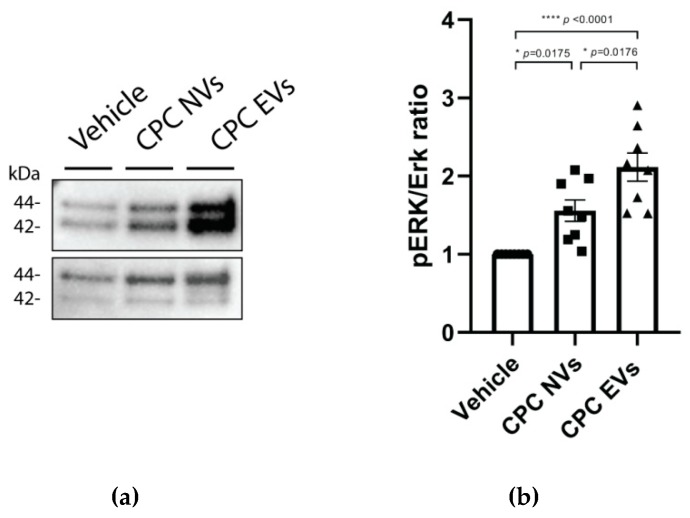
CPC-NVs and CPC-EVs induce phosphorylation of ERK1/2: (**a**) Protein levels of pERK 1/2 and ERK1/2 determined by western blot. (**b**) Quantification of ratio phosphorylated ERK1/2 to ERK1/2 level. Mean results of at least six independent experiments ± SEM.

We have previously used CPC-EVs-induced ERK1/2 phosphorylation in HMEC-1 as a functional outcome measurement [28]. Similar to CPC-EVs, CPC-NVs induced phosphorylation of ERK1/2, as determined by western blot analyses (Figure 5a) and quantified using ImageJ (Figure 4a). These observations show that CPC-NVs, similar to EVs, can activate intracellular pro-survival signaling pathways.

## 4. Discussion

EVs have gained increasing attention over the years for their application as either a drug delivery system or as cell-free therapeutic. However, despite this potential, EVs′ clinical translation is limited by a lack of efficient, scalable, and cost-effective production methods. In this study, we aimed to investigate the biofabrication of NVs as an alternative production method to make EV mimetics. 

NV biofabrication is based on the spontaneous self-assembly of phospholipids, which are abundantly present in the cell membrane [29,30]. Phospholipids are amphiphiles, i.e., they have a hydrophobic tail and hydrophilic headgroup, which enables the cell membrane components to self-assemble into vesicles in an aqueous solution via minimalization of line energy along the free edge of membrane patches. As a result, planar bilayer membrane curves into a spherical cap, ultimately resulting in a closed nanovesicle [29,30]. We hypothesized that NV biofabrication, during which the released cell membranes will self-assemble into cell-derived nanovesicles while encapsulating the released cytosolic material during the self-assembly process, is a more efficient process than EV production to create EV mimetics. 

In this study, we demonstrated that NV biofabrication is a scalable, time-efficient, and cost-effective method capable of yielding cell-derived nanosized vesicles in higher quantities when compared to EV isolation from the same cell density (Figure 2e,f). Furthermore, NVs are produced and purified in a more time-efficient manner compared to EVs (Figure 2g). For both vesicle productions, the most time-consuming step is for cells to reach the desired cell density. However, the upscaling of cells for EV production is more labor-intensive and cost-ineffective compared to production for NVs by biofabrication. After the cell growth phase, vesicle production is the next rate-determining step. When cells are cultured without FBS, EVs are typically isolated after 24–72 h.

In contrast, NVs can be produced within 2 h and when needed once the desired cell density is reached. This convenience makes NVs fabrication a faster alternative to obtain nanosized vesicles with EV-like size and morphology. Additionally, NV biofabrication is a more cost-effective method, since the production phase requires neither additional culture medium nor labor-intensive efforts to isolate and prepare EVs prior to SEC purification. These observations together demonstrate that the generation of NVs is a faster and therefore more efficient method for preparing cell-derived nanovesicles, as compared to the production of natural EVs.

When CPC-NVs were stored at −80 °C, we observe a small increase in size and decrease in concentration. These observations may be explained by NVs′ membrane fusion or adherence to the Eppendorf tubes. Hence, NV storage and stability need to be further characterized. Additionally, the current understanding of EVs storage and stability remains unclear. Hence, EVs and NVs storage and preservation need to extensively investigated in future studies for their therapeutic application [31]. 

NVs have a comparable size distribution (~50–200 nm) and morphology as EVs. Interestingly, while CPC-NVs are enriched for flotillin-1 and calnexin, CPC-EVs are enriched in EV protein markers CD63, CD81, and Alix. NV enrichment of flotillin-1 and calnexin and not with endosomal proteins suggests that CPC-NVs are derived from plasma- and endoplasmic reticulum membranes while CPC-EVs may to a large extent be derived from endosomes. Thus, CPC-EVs and CPC-NVs are two different types of vesicles. These observations are in contradiction with previous studies which show that U937- and adipose stem cell-derived nanovesicles, produced via spin-cups with SEC purification or microfilters followed by centrifugation and Opti-Prep gradient purification, respectively, are enriched with tetraspanin- and multivesicular body proteins [21,32].

Furthermore, the study of Park et al. (2019) demonstrated that mesenchymal stromal cell-derived nanovesicles are enriched with CD81, flotillin-1, and β-actin, while we only found CPC-EVs to be enriched with CD81 [33]. Taken together, these contradictions may indicate that the production of alternative cell-derived nanovesicles is dependent on cell type, production or purification methods. However, to assess the exact difference between NVs and EVs, in-depth proteomics and RNAomics would provide more insight into the differences or similarities between these vesicles. Nevertheless, NVs can be internalized by various cell lines in vitro, suggesting that the lack of the investigated protein markers does not affect the ability of cells to internalize such vesicles. 

EVs deliver cytosolic content of their donor cells to recipient cells that lead to changes in the behavior of cells [34,35]. We showed that NVs are also capable of delivering functional biological material to recipient cells, which induces a cellular activation. Yet, the efficiency of functional transfer via NVs and EVs remains to be determined. Nonetheless, eGFP+ reporter cells are observed when treated with Cre+-EVs and Cre+-NVs derived from different Cre+ donor cells. Importantly, we used donor cells stably expressing Cre, allowing us to study only cell-derived vesicle-mediated transfer of Cre cytosolic RNA (functional RNA) and protein, without the interference of lipid-based carriers that may end up in NVs/EVs and affect their behavior. [36,37,38,39]. 

The observation of eGFP+ reporter cells demonstrates NVs′ potential to be developed as a potential drug delivery system for biotherapeutics produced in cells. Additionally, the cell membrane of the cells used for NV biofabrication can be modified using molecular engineering techniques to express surface molecules. Consequently, these engineered nanovesicles can mediate a specific biological function or neutralize active biological molecules in circulation. Also, the cell membrane can be engineered to contain ligands that recognize specific targets or tissue [40]. Thus, generating NVs from these cell lines could enable NVs to facilitated targeted delivery [1]. Taken together, biofabrication of NVs combined with molecular engineering cells to contain surface proteins or targeting ligand opens up a wide variety of possibilities to use NVs for (targeted) delivery platforms.

Finally, functional assessment of CPC-NVs and CPC-EVs demonstrated that both types of vesicles are capable of activating pro-survival pathways. However, more in vitro functional assays, e.g., apoptosis or proliferation assays, as well as in vivo studies in myocardial infarct mouse models, are required to establish the therapeutic value of CPC-NVs as an alternative for CPC-EVs in regenerative medicine. Furthermore, there remains a lack of understanding regarding the stability and safety of EVs and NVs. Consequently, subsequent studies should be done to address CPC-EVs and CPC-NVs′ stability, toxicity, and potential immunogenicity [18].

## 5. Conclusions

In the present work, we demonstrated that biofabrication of NVs is a scalable, time-efficient, and cost-effective production method to yield more nanoparticles from the same cell density when compared to EVs. Furthermore, we show that CPC-NVs and CPC-EVs share a comparable size and morphology. However, we also show that they are two different types of vesicles regarding their composition of their membrane markers. Yet, NVs can be taken up by hfCF, CPC, and endothelial cells. Finally, we observed the functionality of NVs in delivering Cre-recombinase mRNA or protein as well as activating ERK1/2 signaling. Taken together, we present here an effective method to produce cell-derived nanovesicles with similar functional properties as EVs. Therefore, NVs hold the potential to be employed as a therapeutic agent in regenerative medicine. Additionally, biofabrication of NVs gives rise to new opportunities to create targeted drug delivery systems by functionalizing cell membranes or therapeutic loading cargo inside NVs. 

## Supplementary Materials

The following are available online at https://www.mdpi.com/2073-4409/8/12/1509/s1, Figure S1: An 11-fold higher cell density is needed for EVs production compared to NV biofabrication to yield the same amount of particles, Figure S2: Total number of NVs recovered after SEC is dependent on initial cell density, Figure S3: Characterization of Cre+-EVs and Cre+-NVs, Figure S4: Characterization of CPC-NVs stored at −80 °C over time from two individual isolations.
