# Supplementary material for: Biofabrication of Cell-Derived Nanovesicles: A Potential Alternative to Extracellular Vesicles for Regenerative Medicine
Source: Cells. 2019 Nov 25;8(12):1509. doi: 10.3390/cells8121509 (PMC6952804; doi:10.3390/cells8121509)
Supplement: Supplementary file 1 [file cells-08-01509-s001.zip › cells-645304-proof-SM/cells-645304-supplementary/Supplementary files_Cells_Revised/Supplementary files_Cells_Revised.docx]

**Supplementary files**

**
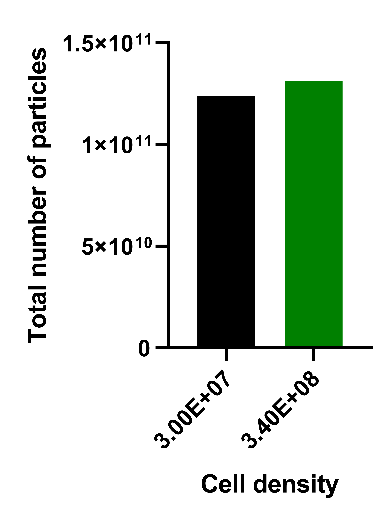
**

**Figure S1. An 11-fold higher cell density is needed for EVs production compared to NV biofabrication to yield the same amount of particles.** NTA shows to gain a comparable total amount of particles for CPC-NVs and -EVs, biofabrication only requires an initial cell density of 3 · 10^7^  of CPC while CPC-EVs production requires an initial cell density of 3.4 · 10^8^  CPC cells. Black = CPC-NVs and green = CPC-EVs.

**
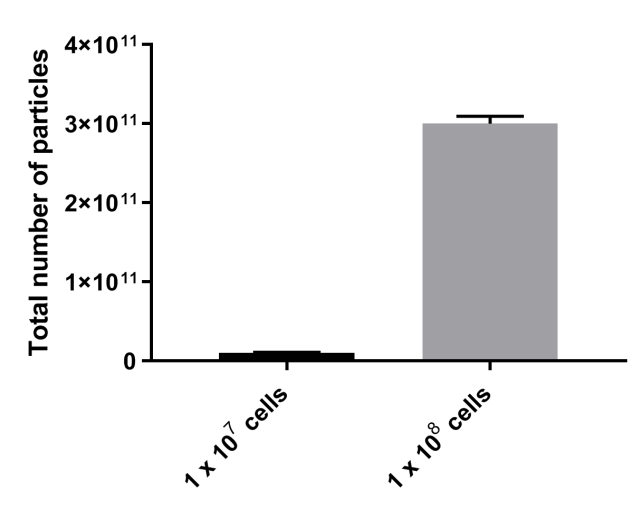
**

**Figure S2. The total number of NVs recovered after SEC is dependent on initial cell density.** NVs production with an initial cell density of 1 · 10^7^ or 1 · 10^8^ HEK293FT cells influenced the total number of NVs recovered after SEC.

**
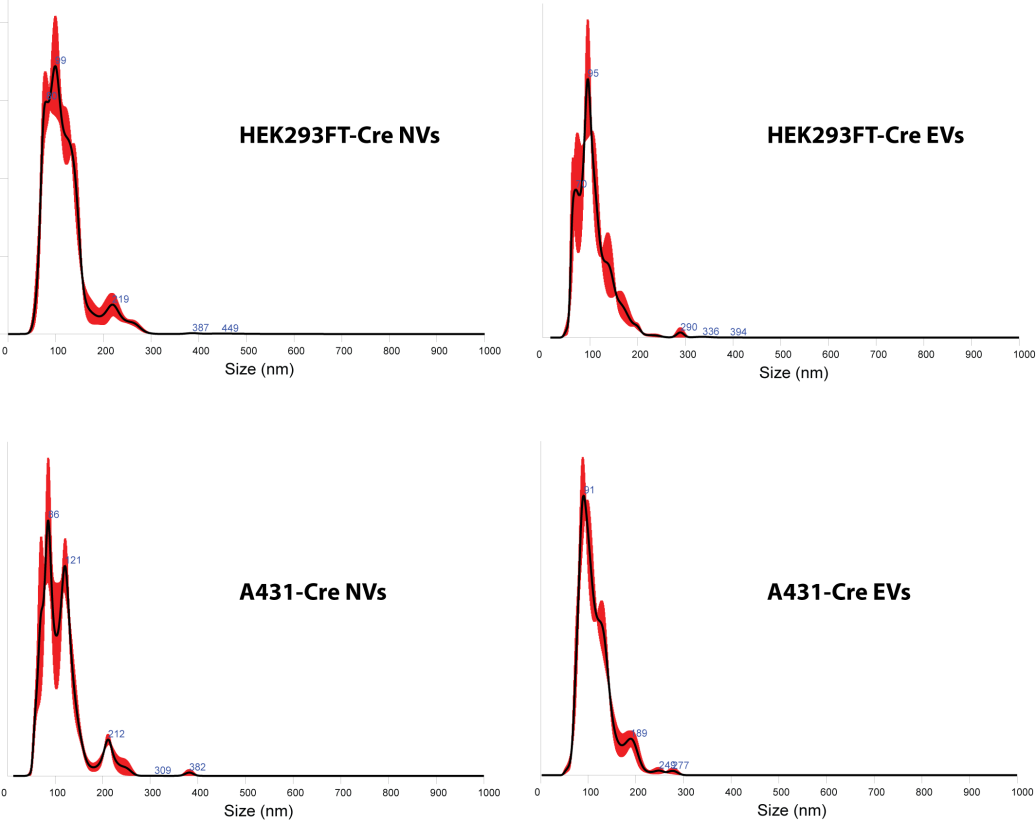
**


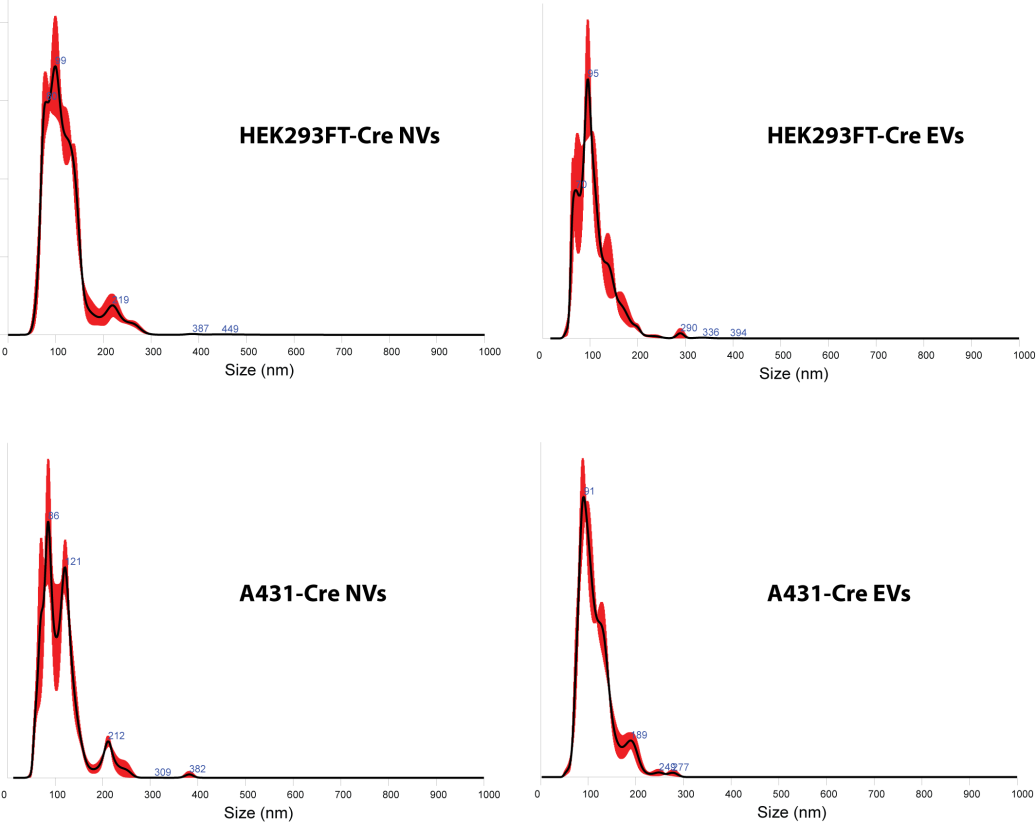


**(a)**

**(b)**

**Figure S3. Characterization of Cre+-EVs and Cre+-NVs** (a**)** NTA of HEK293FT-Cre derived –NVs and EVs and (b) A431-Cre derived –NVs and EVs show diameter between ~50 and 200 nm.

1. **(b) (c)**


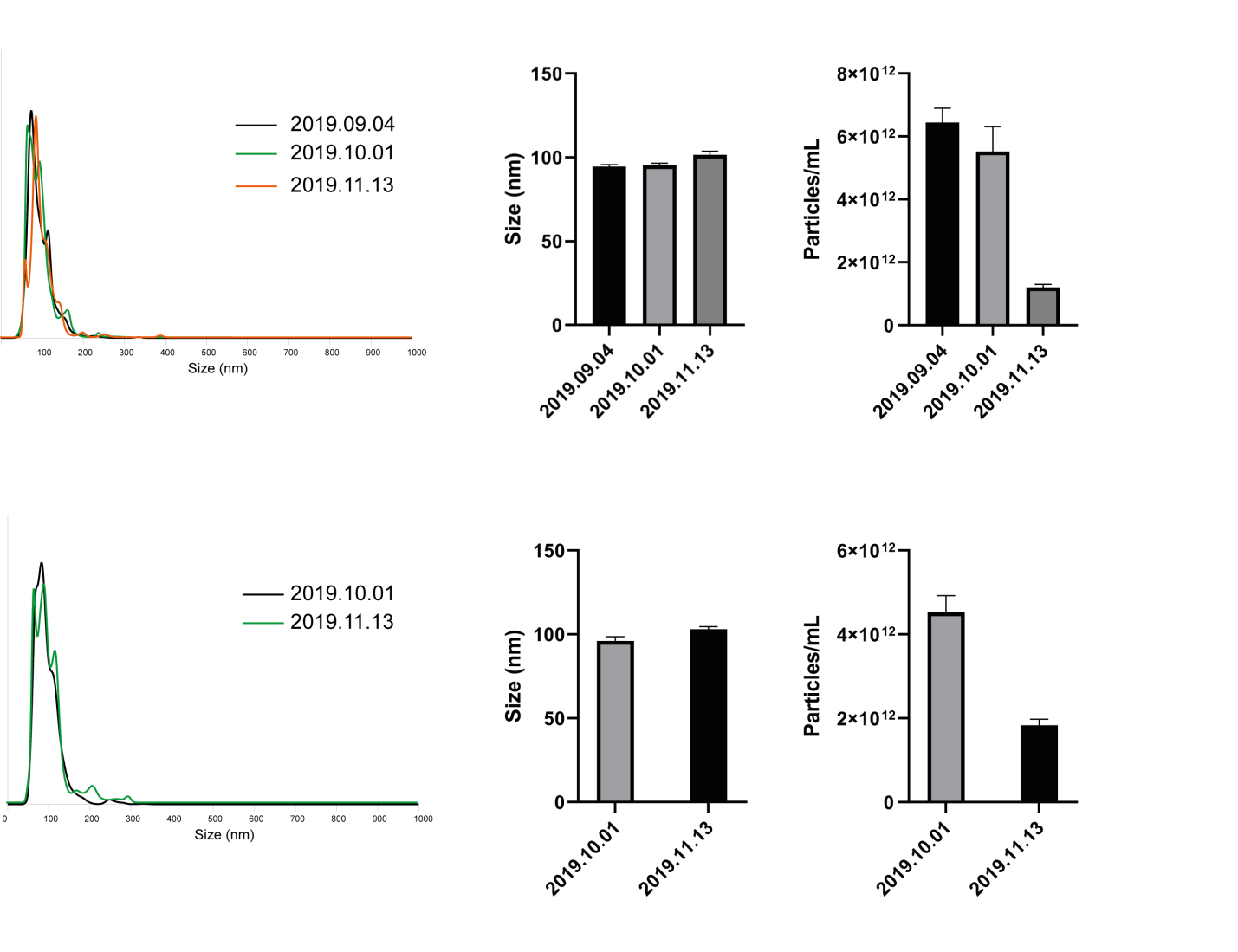


**Figure S4. Characterization of CPC NVs stored at -80°C over time from two individual isolations.** NTA shows that the size distribution of CPC-NVs (a) remain similar over time with a (b) average diameter of ± 98 nm. (c) The number of particles per sample decreases over time, this observation may be explained by adherence of the CPC-NVs to the Eppendorf’s surface.
