# Supplementary figures and images for: Biofabrication of Cell-Derived Nanovesicles: A Potential Alternative to Extracellular Vesicles for Regenerative Medicine
Source: Cells. 2019 Nov 25;8(12):1509. doi: 10.3390/cells8121509 (PMC6952804; doi:10.3390/cells8121509)

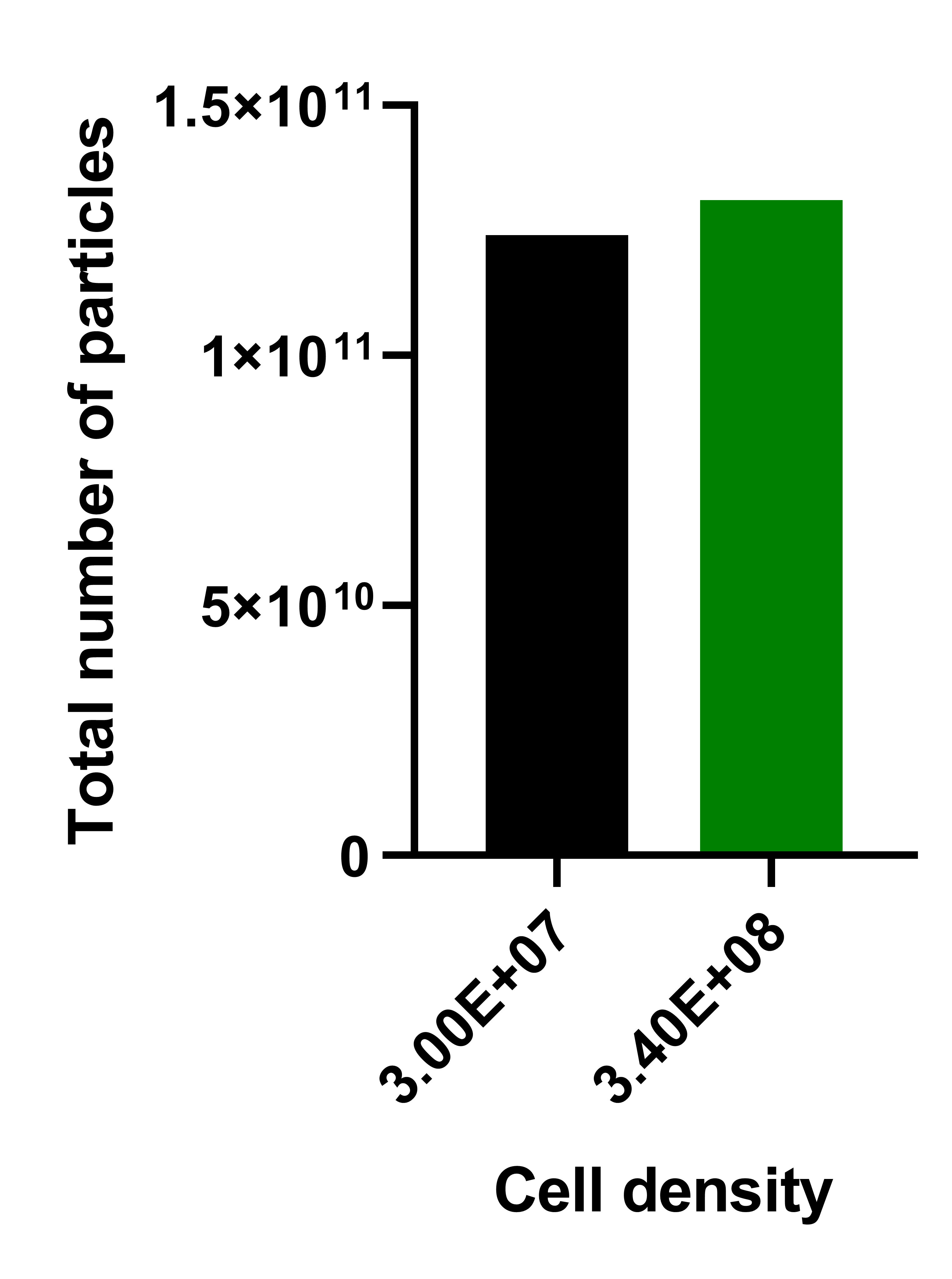

Supplement: Supplementary file 1 [file cells-08-01509-s001.zip › cells-645304-proof-SM/cells-645304-supplementary/Supplementary files_Cells_Revised/Fig S1.tif]

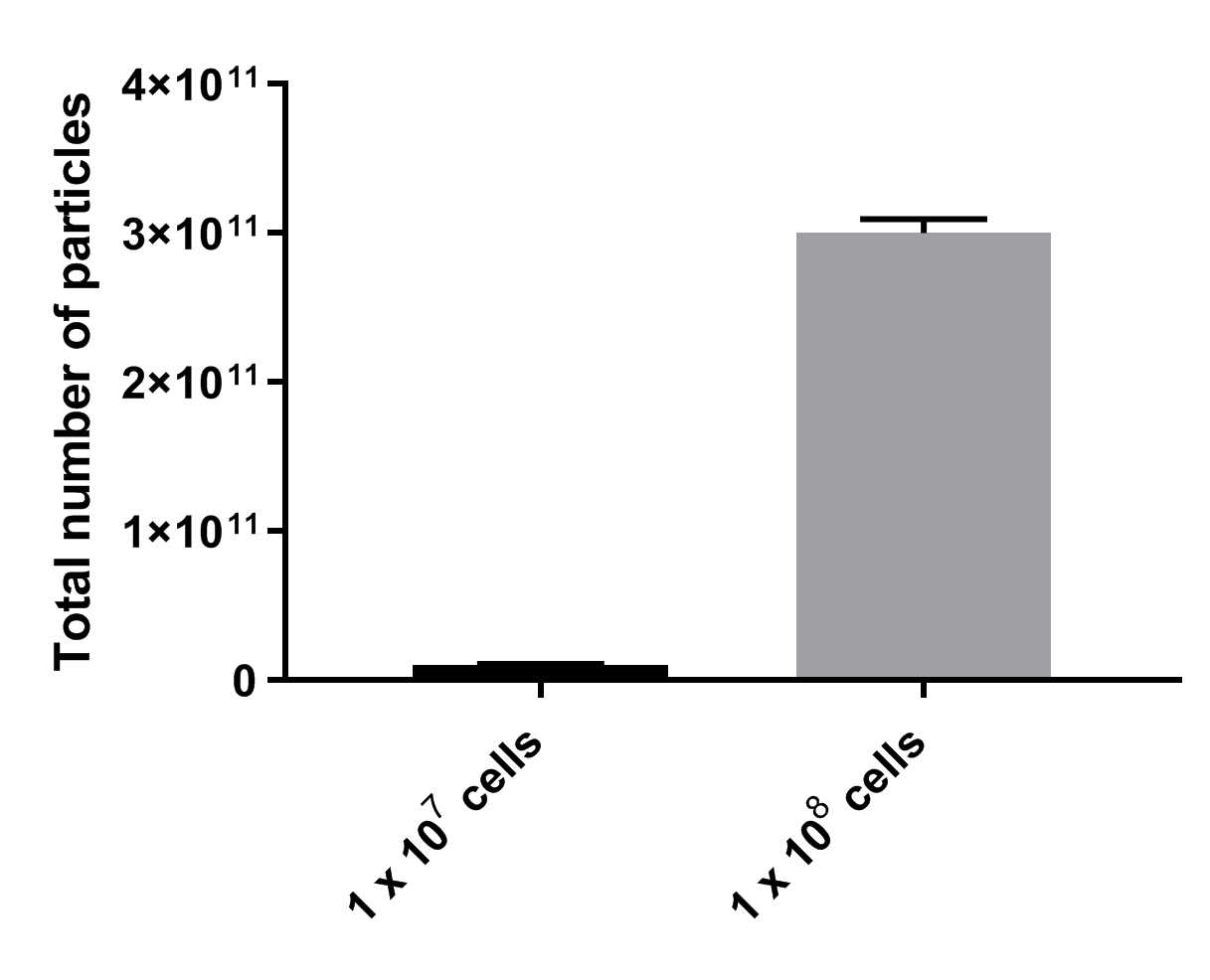

Supplement: Supplementary file 1 [file cells-08-01509-s001.zip › cells-645304-proof-SM/cells-645304-supplementary/Supplementary files_Cells_Revised/Fig S2 Concentration particles_mL 10^7 and 10^8.tif]

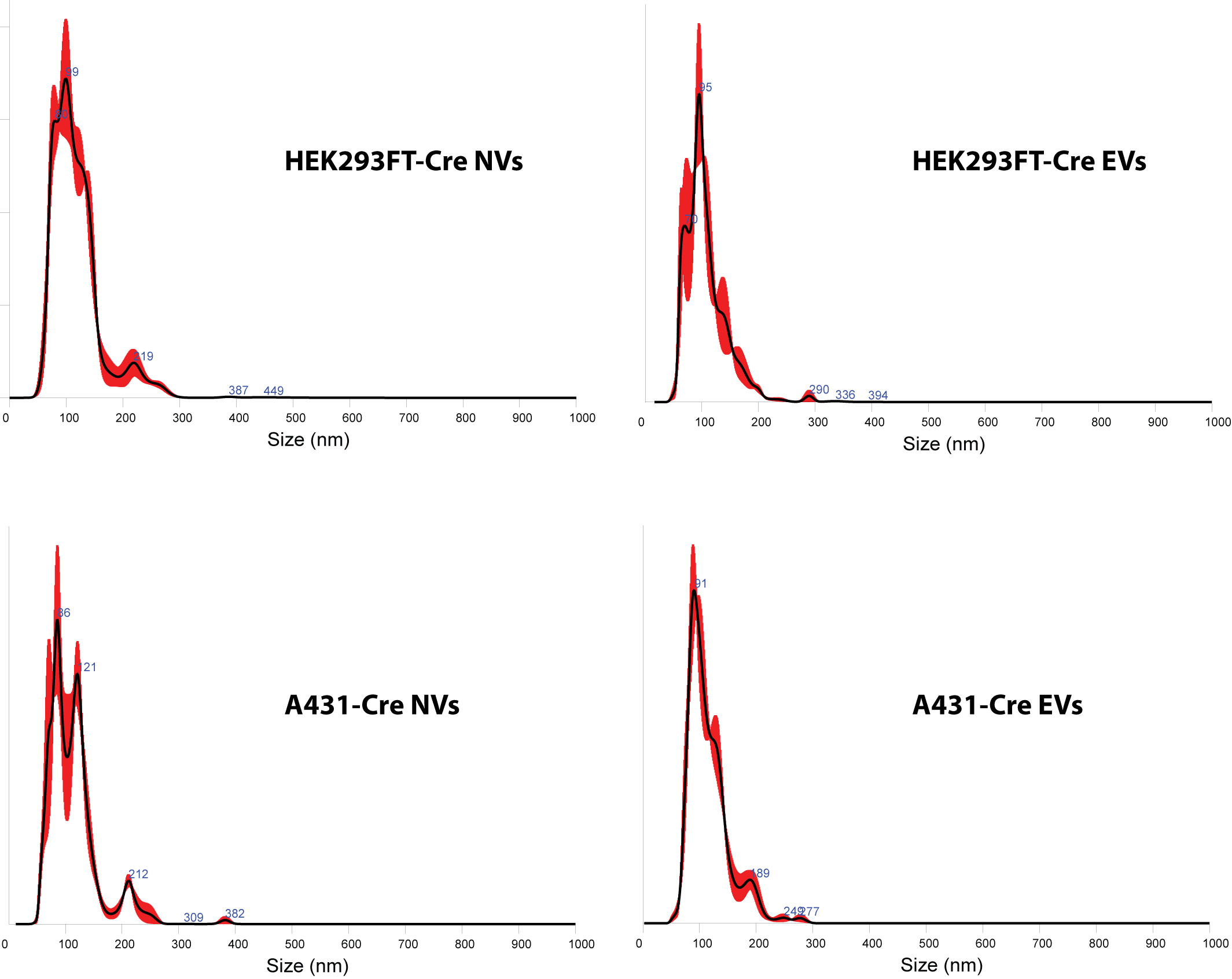

Supplement: Supplementary file 1 [file cells-08-01509-s001.zip › cells-645304-proof-SM/cells-645304-supplementary/Supplementary files_Cells_Revised/Fig S3 NTA Cre NVS and EVs.tif]

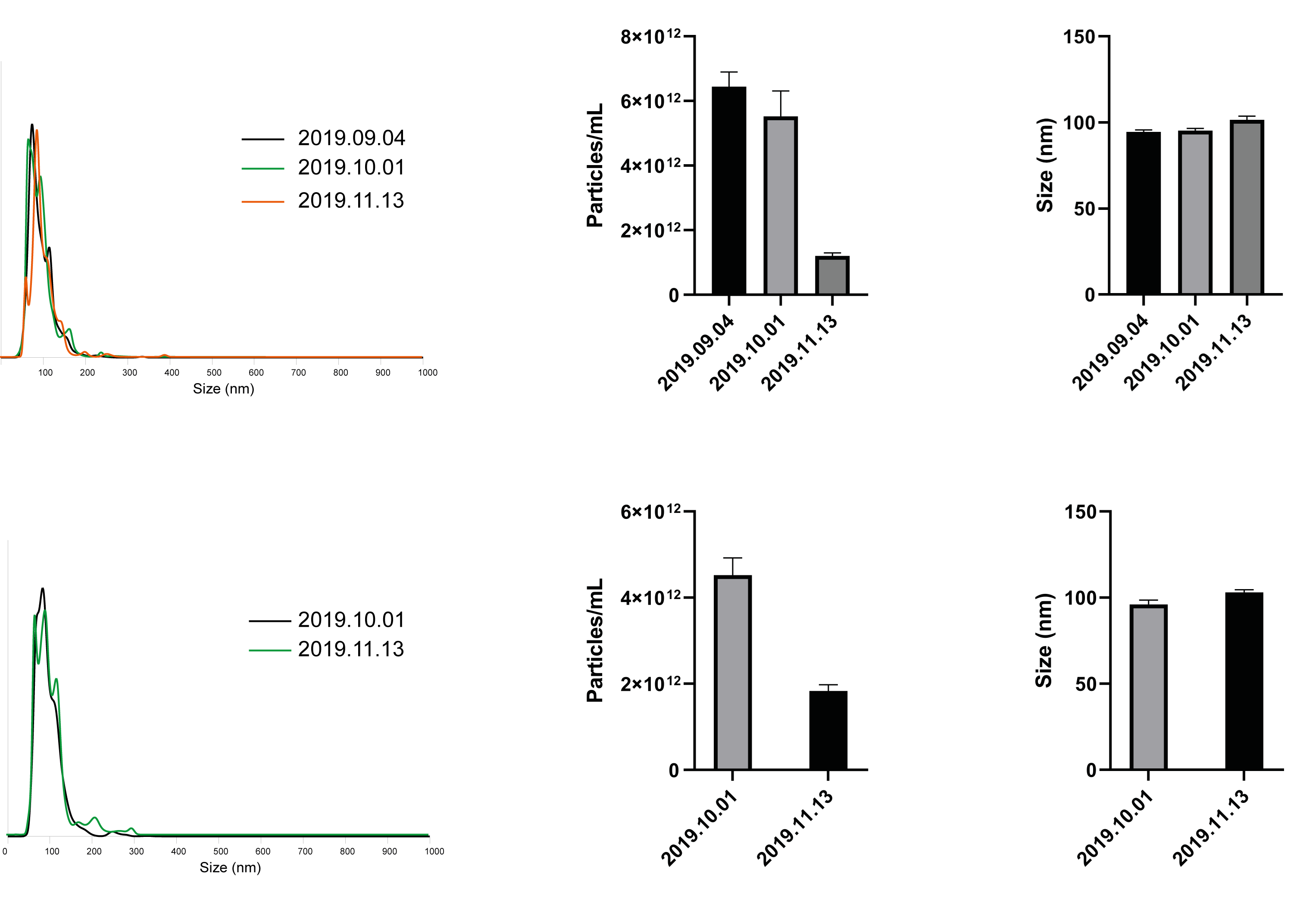

Supplement: Supplementary file 1 [file cells-08-01509-s001.zip › cells-645304-proof-SM/cells-645304-supplementary/Supplementary files_Cells_Revised/Fig S4.tif]
